# Screening for Cognitive Dysfunction Using the Rowland Universal Dementia Assessment Scale in Adults With Sickle Cell Disease

**DOI:** 10.1001/jamanetworkopen.2021.7039

**Published:** 2021-05-13

**Authors:** Stéphanie Forté, Florence Blais, Mathias Castonguay, Nafanta Fadiga, Mireille Fortier-St-Pierre, Maryline Couette, Richard Ward, Sébastien Béland, Melanie Cohn, Denis Soulières, Kevin H. M. Kuo

**Affiliations:** 1 Division of Medical Oncology and Hematology, Department of Medicine, University Health Network (UHN), Toronto, Ontario, Canada; 2 Division of Hematology, Department of Medicine, University of Toronto, Toronto, Ontario, Canada; 3 Division of Medical Oncology and Hematology, Department of Medicine, Centre Hospitalier de l'Université de Montréal, Montréal, Québec, Canada; 4 Faculty of Medicine, Laval University, Québec City, Québec, Canada; 5 Faculty of Arts and Science, Queen’s University, Kingston, Ontario, Canada; 6 Neurorehabilitation Department, Albert Chenevier Hospital, Henri Mondor University, Créteil, France; 7 Sickle Cell Referral Center-Unité des Maladies Génétiques du Globule Rouge, Université Paris-Est Créteil, Centre Hospitalier Universitaire Henri Mondor, Assistance Publique–Hôpitaux de Paris, Créteil, France; 8 Département d'Administration et Fondements de l'Éducation, Faculté des Sciences de l'Éducation, Université de Montréal, Montréal, Québec, Canada; 9 Krembil Research Institute, UHN, Toronto, Ontario, Canada

## Abstract

**Importance:**

Adults with sickle cell disease (SCD) disproportionally experience early cognitive decline; however, guidance on the optimal screening strategy for cognitive dysfunction is lacking, and several available tools are biased by language, educational level, socioeconomic status, and race/ethnicity. The Rowland Universal Dementia Assessment Scale (RUDAS) was specifically designed for cognitive screening in multicultural populations.

**Objective:**

To ascertain the prevalence of suspected dementia in adults with SCD using the RUDAS, and to identify whether age, sex, educational level, several biological variables, and SCD complications were associated with RUDAS scores.

**Design, Setting, and Participants:**

This multicenter, bilingual, cross-sectional study was conducted in 2 SCD comprehensive care centers in Canada (Centre Hospitalier de l’Université Montréal in Montréal and University Health Network in Toronto). Participants were adults aged 18 years or older and were enrolled in the study between July 1, 2018, and July 30, 2019. All outpatients were eligible and offered study participation, unless they had an acute medical condition that required inpatient care or they were unable to follow study instructions.

**Interventions:**

The RUDAS was administered by trained personnel in either French or English, according to the patient’s language preference. A questionnaire on social determinants of health was also administered, and participants underwent screening for anxiety and depression.

**Main Outcomes and Measures:**

Proportion of participants with RUDAS scores that were suggestive of dementia and the RUDAS score. Any score lower than 23 points was suggestive of dementia, a score between 23 and 27 points indicated a possible association with mild neurocognitive disorder, and a score higher than 27 points was normal.

**Results:**

A total of 252 adult patients with SCD were included (136 women [54.0%]; mean [range] age, 34.8 [18-75] years). Overall, 29 patients (11.5%) had RUDAS scores that were suggestive of dementia, and this proportion increased with age (15 [8.7%] in the 18-39 years age group, 10 [14.5%] in the 40-59 years age group, and 4 [36.4%] in the ≥60 years age group). The RUDAS scores were not associated with sex, language, SCD genotype, and SCD complications. The highest level of education was significantly associated with the RUDAS score; however, the association was small (η^2^ = 0.02; 95% CI, 0.00-0.07; *P* = .02). In a multivariable analysis, lower glomerular filtration rate (*r* = 0.40; 95% CI, 0.29-0.50; *P* < .001) and increasing age (*r* = −0.37; 95% CI, −0.47 to −0.26; *P* < .001), but not SCD genotype or disease severity, were associated with lower RUDAS scores.

**Conclusions and Relevance:**

This study found that using the RUDAS revealed a high prevalence of suspected dementia in adult patients with SCD that was associated with worsening kidney function and age. Cognition should be screened in all adult patients with SCD, regardless of age, disease severity, and SCD genotype; further validation of the RUDAS is ongoing.

## Key Points

**Question:**

What is the prevalence of and which factors are associated with suspected dementia in adults with sickle cell disease (SCD)?

**Findings:**

In this cross-sectional study of 252 adult patients with SCD, participants underwent screening using the Rowland Universal Dementia Assessment Scale (RUDAS). The RUDAS scores that were suggestive of dementia were found in 29 patients (11.5%) and were associated with lower kidney function and older age, although they were not associated with sex, SCD genotype, or disease severity.

**Meaning:**

Results of this study suggest that adult patients with SCD may be at an increased risk for dementia and should be offered cognitive screening; the RUDAS is a promising screening instrument that is undergoing further validation.

## Teaser

This cross-sectional study describes the administration and outcome of a short screening tool for assessing executive function, memory, language, and perceptual motor function of adults with sickle cell disease.

## Introduction

Black individuals in the United States are at an increased risk for dementia.^1,2^ Adults with sickle cell disease (SCD), in particular, disproportionally experience cognitive dysfunction.^3^ Sickle cell disease is a genetic condition that occurs in about 1 in every 365 births in the Black or African American population.^4^ Substitution of glutamine by valine in position 6 of the β-globin is responsible for the irreversible sickling of red blood cells, hemolysis, and vaso-occlusion under prolonged deoxygenated conditions. This sickling leads to anemia, pain crises, and systemic vasculopathy that can culminate in stroke, silent cerebral infarcts, reduced cerebral vascular reserve, and cognitive dysfunction.^5,6^ Precise estimates on the prevalence of dementia and mild neurocognitive disorder (NCD) in adults with SCD are lacking, but they could be as high as 33%.^3^ In comparison, 564 000 persons (<2% of the population) in Canada were estimated to live with dementia in 2016.^7^ The cognitive domains associated with SCD are perceptual reasoning, verbal reasoning, executive function, working memory, attention, and processing speed.^8^ These deficits can have implications for quality of life, social functioning, school performance, and employment.^9^

Despite the high prevalence of cognitive dysfunction in adult patients with SCD, no standardized approach for screening exists. In addition, race/ethnicity-based disparities have been associated with delays in diagnosis, more severe presentations, and suboptimal access to care and research.^10^ A brief and easy screening tool is, therefore, urgently needed to aid in the rapid identification of potential dementia as well as to facilitate referral for in-depth neurocognitive evaluation and access to much-needed rehabilitation and assistive services. Existing screening tools, such as the Montreal Cognitive Assessment (MoCA) and Mini-Mental State Examination (MMSE), can be biased by educational attainment, race/ethnicity, socioeconomic factors, and language.^11,12,13,14,15,16^ The Rowland Universal Dementia Assessment Scale (RUDAS) was specifically developed for dementia screening in a multicultural population.^17^ The RUDAS evaluates executive function, memory, language, and perceptual motor function. It was designed to minimize educational bias and can be administered in 6 minutes, even for patients with low literacy. In a meta-analysis of 11 studies (involving 1236 patients) in the general aging population, a low RUDAS score (<23 of 30 points) had a specificity of 85.9% and a sensitivity of 77.2% in identifying dementia.^11^ Compared with the MMSE outcomes, the RUDAS results were not associated with preferred language, age, and educational level.^11^

In this cross-sectional study, our primary aim was to ascertain the prevalence of suspected dementia in adults with SCD using the RUDAS. A secondary aim was to identify whether age, sex, educational level, several biological variables, and SCD complications were associated with RUDAS scores. We hypothesized that the prevalence of suspected dementia in adult patients with SCD estimated by RUDAS is similar to previous studies that used different neurocognitive screening instruments.

## Methods

This study was reviewed and approved by the ethics committee of both participating centers. Written informed consent was obtained from all study participants. We followed Strengthening the Reporting of Observational Studies in Epidemiology (STROBE) reporting guideline.

### Study Design, Setting, and Participants

We conducted a multicenter, bilingual, cross-sectional study in 2 SCD comprehensive care centers in Canada. Participants were drawn from the Centre Hospitalier de l’Université Montréal (CHUM) in Montréal and from the University Health Network (UHN) in Toronto. Between July 1, 2018, and July 30, 2019, we offered study participation to consecutive adult patients (aged ≥18 years) with SCD who attended the outpatient clinic or received blood transfusions at either CHUM or UHN. We excluded participants with an acute medical condition that required inpatient care or with any condition that, according to physician judgment, prevented a valid consent from being obtained and/or compromised the ability of the participant to follow study instructions.

### Data Sources and Measurements

The RUDAS was administered by trained personnel (medical students, nurses, and clinicians) in either French or English, according to the patient’s language preference. A questionnaire on education and employment was administered, and patients’ blood pressure was measured. One of us (S.F.) trained the test administrators in person according to the RUDAS administration guide. Unplanned monitoring visits were performed to verify the quality of test administration. Two of us (S.F. and D.S.) assessed the quality of test scoring.

The RUDAS scores were abstracted from the questionnaires. Demographic data, SCD complications, laboratory results, and radiographic findings were abstracted from the electronic patient record systems at both CHUM and UHN. Abstractors were provided with data collection manuals and training to standardize the data collection process. Calibration was done through regular meetings and ongoing communications.

### Outcomes Definition, Variables, and Sample Size Calculation

The RUDAS scores were defined according to the RUDAS manual for scoring. According to the definitions described by Basic et al,^18^ any score lower than 23 points was suggestive of dementia, and participants with such scores were referred for further neuropsychological testing. Scores higher than 27 points were considered normal. Scores between 23 and 27 points indicated a possible association with mild NCD. Clinical variables collected were age, sex, SCD genotype, α-thalassemia sequence variation status, previous or current blood transfusion, and hydroxyurea therapy. Complications were identified according to the definitions described by Sebastiani et al.^19^ Stroke was confirmed by medical history and imaging. Patients were noted to have dementia, depression, or anxiety disorder only if they were formally diagnosed with this condition by a health care practitioner. The SCD severity score was calculated using the online calculator developed by Sebastiani et al.^19,20^

Socioeconomic factors included mean household income (expressed as a continuous variable), which was estimated from postal codes data on the CensusMapper website, a resource based on the 2016 Canadian census data. Highest level of education was ranked according to the highest completed educational program (did not complete high school; high school diploma or equivalent; apprenticeship or trade certificate; college or other nonuniversity certificate or diploma; <bachelor’s degree–level university certificate; and ≥bachelor’s degree–level university certificate, diploma, or degree). Occupational status was dichotomized as either working and/or studying or not working and not studying. No differentiation was made between part-time vs full-time employment or studies.

The most recent laboratory values of bilirubin, lactate dehydrogenase, hematocrit, reticulocyte count, mean corpuscular volume, fetal hemoglobin fraction, white blood cells, and glomerular filtration rate (GFR) within 1 year of enrollment were collected. Glomerular filtration rate was estimated using the Modification of Diet in Renal Disease Study equation. To ensure that laboratory parameters were not confounded by acute medical events, such as painful episodes, we used only the laboratory results from more than 3 weeks from an emergency department or inpatient admission.

An estimated sample size of 255 participants (of whom 85 [33.3%] were estimated to have cognitive dysfunction^3^) was required, assuming an area under the curve of 0.9 for the RUDAS and an SE of 0.02348. This SE was the most conservative estimate derived from all previous studies on the RUDAS that provided an SD.^21,22,23^

### Statistical Analysis

Summary statistics of continuous variables were presented as either a mean (SD) or a median (range), whichever was appropriate. For categorical variables, frequencies were calculated. Mean RUDAS scores were stratified by age group (18-39, 40-59, and ≥60 years), and the proportions of participants with RUDAS scores lower than 23 and higher than 27 were calculated for each stratum.

The RUDAS score was standardized by subtracting every score from the mean and dividing by the SD. Skewness was assessed to be moderate. First, we investigated the association between these RUDAS *z* scores and 29 variables of interest. For discrete variables, we used independent unpaired, 2-tailed *t* test or 1-way analysis of variance. Effect sizes were quantified using Cohen *d* and η^2^. For continuous variables, we computed Pearson correlations. Multiple imputation of all missing variables was performed using the mice package in R (R Foundation for Statistical Computing).^24^ Five iterations were used. Holm correction was used to correct for multiplicity.

Second, we produced linear multiple regression to identify the independent variables associated with the RUDAS *z* scores. Twelve independent variables were chosen a priori: age, sex, highest level of education, mean household income, SCD genotype, hematocrit, GFR, reticulocyte count, stroke, depression, anxiety, and pain. Statistical significance was defined as 2-sided *P* < .05. Multicollinearity was assessed using variance inflation factor (VIF). Cohen *f*^2^ was reported to measure the effect size of the regression model. Statistical analyses were performed with R.^24^

## Results

### Participants

Between July 1, 2018, and July 30, 2019, we enrolled 252 participants, of whom 92 were from CHUM and 160 were from UHN. Of these patients, 136 were women (54.0%) and 116 were men (46.0%), with a mean (range) age of 34.8 (18-75) years. Overall, we offered study participation to a total of 260 patients; 1 candidate was excluded because of inability to follow study instructions and 7 candidates declined the invitation. All participants at UHN chose the English questionnaires, whereas 85 participants (94.6%) at CHUM preferred the French questionnaire.

A significant proportion of patients (69 [27.4%]) was in the 40 to 59 age group (Table 1). The SCD genotype distribution was as follows: sickle cell anemia (SS and Sβ^0^) in 152 (60.4%), SC in 79 (31.3%), Sβ^+^ in 9 (3.6%), and other sickle genotypes in 12 (4.8%). Two patients (0.8%) had a clinical diagnosis of dementia, and 24 patients (9.5%) had a stroke history. Pain (192 [76.2%]) and acute chest syndrome (110 [43.7%]) were the most frequent complications. The median (interquartile range [IQR]) Sebastiani SCD severity score was 0.17 (0.09-0.24) points, an estimated 17% chance of death within 5 years. Overall, 177 patients (70.2%) were on a disease-modifying therapy (hydroxyurea and/or regular transfusions).

**Table 1.** Baseline Data for Study Participants

Characteristic	No. (%) (N = 252)
Biological factors	
Age, mean (range), y	34.8 (18-75)
Distribution by age group, y	
18-39	172 (68.3)^a^
40-59	69 (27.4)^a^
≥60	11 (4.4)^a^
Sex	
Male	116 (46.0)
Female	136 (54.0)
SCD genotype	
SS or Sβ^0^	152 (60.4)^a^
SC	79 (31.3)^a^
Sβ^+^	9 (3.6)^a^
Other sickle genotype	12 (4.8)^a^
α-Thalassemia sequence variation	34 (13.5)
Language and social determinants of health	
Language	
French	87 (34.5)
English	165 (65.5)
Highest level of education^b^	
Did not complete high school	19 (7.7)
High school diploma or equivalent	83 (33.5)
Apprenticeship or trade certificate	3 (1.2)
College or other nonuniversity certificate or diploma	74 (29.8)
<Bachelor’s degree–level university certificate	1 (0.4)
≥Bachelor’s degree–level university certificate, diploma, or degree	68 (27.4)
Mean household income, (SD), CAD$^b^	58 512 (20 691)
Occupational status^b^	
Working and/or studying	212 (84.1)
SCD-related complications	
History of stroke	24 (9.5)
SCD-related pain	192 (76.2)
History of acute chest syndrome	110 (43.7)
Sebastiani SCD severity score, median (IQR), points	0.17 (0.09-0.24)
Comorbid conditions	
Dementia	2 (0.8)
Depression	20 (7.9)
Anxiety	15 (6.0)
Systolic blood pressure, mean (SD), mm Hg	118 (16)
Laboratory values^b^	
Hematocrit, mean (SD), L/L	0.29 (0.05)
Lactate dehydrogenase, median (IQR), U/L	312 (233-416)
Bilirubin, median (IQR), mg/dL	1.7 (1.2-2.7)
Reticulocyte count, median (IQR), %	5.7 (3.4-8.8)
Fetal hemoglobin fraction, median (IQR), %	4.0 (1.2-14.4)
Glomerular filtration rate, median (IQR), mL/min/1.73 m^2^	118 (100-120)
Disease modifying therapies	
Hydroxyurea	123 (49.0)
Regular exchange transfusion	61 (24.2)
None	75 (29.8)

Abbreviations: CAD$, Canadian dollar; IQR, interquartile range; SCD, sickle cell disease.

SI conversion factors: To convert bilirubin to micromoles per liter, multiply by 17.104; lactate dehydrogenase to microkatals per liter, multiply by 0.0167.

^a^ Percentages do not sum up to 100% because of rounding.

^b^ Missing data: highest level of education, 1.6%; mean household income, 1.2%; occupational status, 0.8%; systolic blood pressure, 4.8%; hematocrit, 6.7%; lactate dehydrogenase, 21.0%; bilirubin, 8.7%; reticulocyte count, 17.9%; fetal hemoglobin fraction, 10.3%; glomerular filtration rate, 9.9%.

### Outcomes

The mean RUDAS score was 25.9 (2.9) points, and the median (IQR) RUDAS score was 26 (24-28) points (Table 2). French-speaking cohorts had a mean (SD) RUDAS score of 26.2 (2.9) points, and the English-speaking cohorts had a similar mean (SD) score of 25.9 (2.9) points. The mean RUDAS scores decreased significantly with increasing age (26.5 [2.5] points in the 18-39 years age group vs 25.2 [3.1] points in the 40-49 years age group vs 22.7 [3.4] points in the ≥60 years age group). Suspected dementia (RUDAS score <23 of 30 points) was found in 29 patients (11.5%). The frequency of a RUDAS score lower than 23 was 15 patients (8.7%) in the 18 to 39 years age group, 10 patients (14.5%) in the 40 to 59 years age group, and 4 patients (36.4%) in the 60 years or older group. A total of 74 participants (29.4%) aged 18 to 39 years had a normal RUDAS score (>27 points), but this was the case for only 20 participants (7.9%) aged 40 to 59 years and none in the older group. Most participants (129 [51.2%]) had scores between normal and suspected dementia (23-27 points).

**Table 2.** Rowland Universal Dementia Assessment Scale (RUDAS) Scores Stratified by Age Group

RUDAS score	No. (%)			
	18-39 y (n = 172)	40-59 y (n = 69)	≥60 y (n = 11)	Overall (n = 252)
Mean (SD), points	26.5 (2.5)	25.2 (3.1)^a^	22.7 (3.4)^a^	25.9 (2.9)^b^
<23	15 (8.7)	10 (14.5)	4 (36.4)	29 (11.5)
23-27	83 (61.9)	39 (77.6)	7 (63.6)	129 (51.2)
>27	74 (29.4)	20 (7.9)	0	94 (37.3)

^a^ Post hoc Tukey honestly significant difference showed a most significant difference between those in the 18 to 39 years and 60 years or older age groups (*P* < .001). Differences between the 18 to 39 years and 40 to 59 years age groups (*P* = .002) and the 40 to 59 years and 60 years or older age groups (*P* = .02) were also statistically significant.

^b^ Overall significance was *P* < .001, calculated through analysis of variance.

### Exploratory Analyses

#### RUDAS Score and Social Determinants of Health

On univariate analysis (Table 3), sex, study site, language, and mean household income did not correlate with the RUDAS *z* scores, whereas the highest level of education had a small but statistically significant association with the RUDAS *z* scores (η^2^ = 0.02; 95% CI, 0.00-0.07; *P* = .02). The mean difference was 0.8 points higher in those with at least a bachelor’s degree (26.6 [2.4] points) vs those without a bachelor’s degree (25.8 [3.1] points). On post hoc analysis, significant difference in RUDAS performance was observed only between those without any diploma, certificate, or degree and those with at least a bachelor’s degree (24.2 [3.5] points vs 26.6 [2.4] points; post hoc Tukey honestly significant difference [HSD] *P* = .01; all other pairwise comparisons of the mean RUDAS score for each other educational level category had no association).

**Table 3.** Summary of Univariate Analysis of Rowland Universal Dementia Assessment Scale *z* Scores and Other Variables

Variable	No imputation			Multiple imputation		
	Test statistic^a^	*P* value	Effect size^b^ (95% CI)	Test statistic^a^	*P* value	Effect size^b^ (95% CI)
Biological factors						
Sex	*t* = −1.050	.30	Cohen *d* = −0.13 (−0.38 to 0.12)	No missing data		
Age	*r* = −0.37	<.001	*r* = −0.37 (−0.47 to −0.26)			
SCD genotype	*F* = 0.004	.95	η^2^ = 0.00 (0.00 to 0.005)			
α-Thalassemia sequence variation status	*t* = 0.957	.34	Cohen *d* = 0.20 (−0.17 to 0.56)			
Language and social determinants of health						
Site	*F* = 0.625	.43	η^2^ = 0.002 (0.00 to 0.03)	No missing data		
Language	*t* = 0.755	.45	Cohen *d* = 0.10 (−0.16 to 0.36)			
Mean household income^c^	*r* = 0.03	.67	*r* = 0.03 (−0.10 to 0.15)	*r* = 0.03	.67	*r* = 0.03 (−0.10 to 0.15)
Highest level of education^c^	*F* = 6.004	.02	η^2^ = 0.02 (0.00 to 0.07)	*F* = 6.004	.02	η^2^ = 0.02 (0.00 to 0.07)
Occupational status^c^	*F* = 21.93	<.001	η^2^ = 0.08 (0.03 to 0.15)	*F* = 21.65	<.001	η^2^ = 0.08 (0.02 to 0.15)
SCD-related complications						
History of acute chest syndrome	*t* = −1.197	.23	Cohen *d* = −0.15 (−0.40 to 0.10)	No missing data		
Self-reported SCD related pain	*t* = 0.092	.93	Cohen *d* = −0.01 (−0.28 to 0.30)			
Sepsis	*t* = −0.023	.98	Cohen *d* = −0.01 (−0.67 to 0.60)			
Stroke	*t* = −0.376	.71	Cohen *d* = −0.09 (−0.51 to 0.34)			
Sebastiani SCD severity score	*r* = −0.03	.62	*r* = −0.03 (−0.15 to 0.09)			
Comorbid conditions						
Depression	*t* = −1.788	.09	Cohen *d* = −0.31 (−0.77 to 0.15)	No missing data		
Anxiety disorder	*t* = −0.896	.38	Cohen *d* = −0.28 (−0.80 to 0.25)			
Systolic blood pressure^c^	*r* = −0.14	.03	*r* = −0.14 (−0.26 to −0.01)	*r* = −0.13	.05	*r* = −0.13 (−0.25 to −0.00)
Laboratory parameters^c^						
Bilirubin	*r* = 0.09	.20	*r* = 0.09 (−0.40 to 0.21)	*r* = 0.07	.29	*r* = 0.07 (−0.06 to 0.20)
Lactate dehydrogenase	*r* = 0.07	.35	*r* = 0.07 (−0.07 to 0.20)	*r* = 0.09	.22	*r* = 0.09 (−0.06 to 0.24)
Hematocrit	*r* = −0.05	.49	*r* = −0.05 (−0.17 to 0.08)	*r* = 0.04	.49	*r* = −0.04 (−0.17 to 0.08)
Reticulocyte count	*r* = 0.17	<.05	*r* = 0.17 (0.04 to 0.30)	*r* = 0.16	.03	*r* = 0.17 (0.02 to 0.29)
Mean corpuscular volume	*r* = 0.00	.99	*r* = 0.00 (−0.13 to 0.13)	*r* = 0.00	.96	*r* = 0.00 (−0.12 to 0.13)
Fetal hemoglobin fraction	*r* = 0.01	.86	*r* = 0.01 (−0.12 to 0.14)	*r* = 0.03	.67	*r* = 0.01 (−0.10 to 0.14)
White blood cell count	*r* = 0.06	.38	*r* = 0.06 (−0.07 to 0.18)	*r* = 0.06	.39	*r* = 0.06 (−0.07 to 0.18)
Glomerular filtration rate	*r* = 0.40	<.001	*r* = 0.40 (0.29 to 0.50)	*r* = 0.43	<0.001	*r* = 0.43 (0.32 to 0.52)
Disease-modifying therapies						
Previous blood transfusion	*t* = −0.686	.49	Cohen *d* = −0.10 (−0.39 to 0.18)	No missing data		
Hydroxyurea	*t* = 0.57	.57	Cohen *d* = 0.07 (−0.18 to 0.32)	*t* = 0.57	.58	Cohen *d* = 0.06 (−0.13 to 0.31)
Regular exchange transfusion	*t* = −1.24	.22	Cohen *d* = −0.17 (−0.46 to 0.12)	*t* = −1.24	.24	Cohen *d* = −0.17 (−0.45 to 0.12)

Abbreviation: SCD, sickle cell disease.

^a^ For binary variables, results of the *t* test are shown; for categorical variables, results of the *F* tests are shown; for continuous variables, Pearson *r* coefficients are shown.

^b^ For binary variables, effect sizes are reported with the Cohen *d*; for categorical variables, effect sizes are reported with the η^2^ statistics.

^c^ Missing data: highest level of education, 1.6%; mean household income, 1.2%; occupational status, 0.8%; systolic blood pressure, 4.8%; hematocrit, 6.7%; lactate dehydrogenase, 21.0%; bilirubin, 8.7%; reticulocyte count, 17.9%; fetal hemoglobin fraction, 10.3%; glomerular filtration rate, 9.9%.

Similarly, occupational status had a small but statistically significant association with the RUDAS *z* scores (η^2^ = 0.08; 95% CI, 0.03-0.15; *P* < .001). On post hoc analysis, the RUDAS scores in those who were both studying and working were 3.4 points higher or those who were studying were 1.9 points higher (post hoc Tukey HSD *P* = .009) than the scores in those neither studying nor working (post hoc Tukey HSD *P* < .001). Studying and working status was associated with a 2.1-point higher RUDAS score compared with only working (26.6 [2.4] points vs 24.2 [3.5] points; post hoc Tukey HSD *P* = .007; all other pairwise comparisons of the mean RUDAS score for each occupational status category had no association).

#### RUDAS Score and Neurological Comorbidities

The association of a history of dementia diagnosis with the RUDAS score was not measured because only 2 patients with such history were included. A diagnosis of depression or anxiety disorder was not associated with the RUDAS *z* scores (depression: Cohen *d* = −0.31 [95% CI, −0.77 to 0.15; *P* = .09]; anxiety disorder: Cohen *d* = −0.28 [95% CI, −0.80 to 0.25; *P* = .38]). Stroke was not associated with the RUDAS *z* scores (Cohen *d* = −0.09; 95% CI, −0.51 to 0.34; *P* = .71) (Table 3).

#### RUDAS Score, Biological Variables, and SCD Complications

We confirmed that the RUDAS *z* scores decreased significantly with increasing age (age as a continuous variable, *r* = −0.37; 95% CI, −0.47 to −0.26; *P* < .001). The RUDAS *z* scores also decreased significantly with lower GFR (*r* = 0.40; 95% CI, 0.29-0.50; *P* < .001) and decreased more modestly with increasing systolic pressure (*r* = −0.14; 95% CI, −0.26 to −0.01; *P* = .03) and lower reticulocyte count (*r* = 0.17; 95% CI, 0.04-0.30; *P* = .01). The SCD genotype, complications, and severity score did not correlate with the RUDAS *z* scores (Table 3).

#### Multiplicity 

After applying a Holm correction for multiplicity, the associations of the RUDAS *z* scores with age, occupational status, and GFR remained statistically significant. Significance was no longer observed for highest level of education, systolic pressure, and reticulocyte count (eTable 1 in the Supplement).

#### Multiple Linear Regression

On multiple linear regression (Table 4), after adjusting for 12 variables that were chosen a priori, only age (unstandardized RUDAS score estimate, −0.01; 95% CI, −0.02 to 0.00; *P* = .03), highest level of education (unstandardized RUDAS score estimate, 0.09; 95% CI, 0.02-0.16; *P* = .02), and GFR (unstandardized RUDAS score estimate, 0.01; 95% CI, 0.01-0.02; *P* < .001) were independently associated with the RUDAS *z* scores. Our model accounted for 24% of the variability in the RUDAS scores (*R*^2^ = 0.24 [*f*^2^ = 0.32]; adjusted *R*^2^ = 0.20 [*f*^2^ = 0.25]). Collinearity was overall acceptable (VIF range, 1.06-1.70) and was highest for age (VIF = 1.70) and GFR (VIF = 1.58). The result did not differ significantly when missing data were imputed (eTables 2 and 3 in the Supplement).

**Table 4.** Estimated Rowland Universal Dementia Assessment Scale (RUDAS) *z* Scores Using Multiple Regression^a^

Variable	Unstandardized RUDAS score estimate (95% CI)	SE	Standardized RUDAS score estimate	*t* Value	*P* value
(Intercept)	−1.07 (−2.31 to 0.18)	0.631	0.000	−1.689	.09
Age	−0.01 (−0.02 to 0.00)	0.006	−0.170	−2.147	.03
Sex	0.21 (−0.06 to 0.47)	0.134	0.106	1.557	.12
Highest level of education	0.09 (0.02 to 0.16)	0.036	0.154	2.435	.02
Mean household income	0.00 (0.00 to 0.00)	0.000	0.063	1.000	.32
SCD genotype	−0.01 (−0.13 to 0.12)	0.062	−0.005	−0.081	.94
Stroke	−0.05 (−0.45 to 0.36)	0.206	−0.015	−0.239	.81
Depression	0.28 (−0.16 to 0.73)	0.227	0.083	1.244	.22
Anxiety	−0.06 (−0.59 to 0.46)	0.266	−0.016	−0.236	.81
Pain	−0.16 (−0.45 to 0.12)	0.145	−0.070	−1.125	.26
Hematocrit	−1.53 (−4.06 to 1.00)	1.282	−0.085	−1.195	.23
GFR	0.01 (0.01 to 0.02)	0.003	0.323	4.221	<.001
Reticulocyte count	0.28 (−0.01 to 0.57)	0.148	0.125	1.881	.06

Abbreviations: GFR, glomerular filtration rate; SCD, sickle cell disease.

^a^ Overall estimate of the multiple regression model: *F*_(12, 205)_ = 5.489, *P* < .001; *R^2^* = 0.24 (*f*^2^ = 0.32); adjusted *R^2^* = 0.20 (*f*^2^ = 0.25).

## Discussion

To our knowledge, this study analyzed the largest cohort of patients with SCD who underwent cognitive screening. Of these patients, 11.5% had RUDAS scores that were suggestive of dementia. This proportion increased with age, but sex and language were not associated with the results. Glomerular filtration rate, but not SCD genotype, complications, or disease severity score, was associated with the RUDAS scores.

Using a stringent RUDAS score cutoff lower than 23, the study found a lower prevalence of suspected cognitive dysfunction than that reported in a few published studies that screened for cognitive dysfunction in adult patients with SCD.^12,13,25,26^ However, comparisons between these studies must be made with caution because different definitions of cognitive dysfunction were used. A cross-sectional study that used the MMSE reported a 25% prevalence of suspected dementia.^25^ The MoCA has also been used in SCD trials, as reported in 3 separate single-center studies in the US with 100 patients, in Brazil with 56 patients, and in France with 45 patients.^12,13,26^ Use of the MoCA resulted in 46% of participants in the US, 82% of participants in Brazil, and 64% of participants in France attaining scores that were lower than the cutoff threshold for mild NCD, a less severe NCD that did not impede independence in performing instrumental activities of daily living.^12,13,26^ If patients with the RUDAS scores of 23 to 27 points were included, the prevalence of suspected cognitive dysfunction would reach 63%, which is similar to the rate in previous studies. Although this range of the RUDAS scores has been correlated with mild NCD in 1 study,^18^ we are cautious to interpret it because precise cutoffs for mild NCD have not been formally validated. Similar to other cognitive screening instruments, such as the MoCA, the RUDAS needs SCD-specific thresholds for scores suggestive of dementia and mild NCD.

We cannot overlook that educational attainment played a role in the RUDAS, similar to other screening tools, given that we observed an association between highest level of education and the RUDAS *z* scores in the univariate analysis but not in the multivariable analysis or after correction for multiplicity. A direct comparison of the RUDAS scores to the MoCA scores in 45 patients with SCD in France suggested that educational attainment was less of a factor in the RUDAS than in the MoCA.^26^ Adjustment for educational level removed this association with the RUDAS but not with the MoCA.^26^ It is also possible that the findings of the present study reflect a protective feature of education for dementia risk, as is the case in the general population.^27^

The exploratory finding that GFR and age are potential risk factors for cognitive dysfunction in SCD is in line with the results of 2 studies that used the MoCA.^12,13^ The robust association of the RUDAS scores with age and kidney function mirrors previous findings in the general population, in which they were some of the strongest risk factors of mild NCD and dementia.^28,29^ The precise mechanisms involved are unknown. One potential mediator could be high blood pressure, whereby chronic vascular injury develops concurrently in both the brain and the kidney. In addition, impaired blood flow autoregulation is a common pathophysiological mechanism by which end-organ damage is thought to occur and merits further exploration.^30,31,32,33,34^ Although SCD genotype is often considered a milder form of SCD because of its more indolent disease course, including a lower risk for chronic kidney disease,^35^ it was not protective against cognitive dysfunction in this study cohort. This finding suggests that patients with all SCD genotype should receive the same attention for neurocognitive screening.

In this study, stroke, depression, anxiety, and pain were not associated with the RUDAS scores, possibly because of the limited number of events. In addition, the prevalence of anxiety and depression was most likely underestimated because no formal systematic screening was performed before this study. The lack of association of the RUDAS score with the disease severity score could be attributed to the limited validity of the Sebastiani SCD severity score in more contemporaneous cohorts given that the primary cohort in which the SCD severity score was constructed was recruited in the 1970s to 1980s for the Cooperative Study of Sickle Cell Disease.^11^ It is also possible that the risk factors for mortality are different from the risk factors for dementia.

Findings of this study are in line with the recommendation by the American Society of Hematology.^36^ Although the RUDAS appears to be an appealing tool for dementia screening among multilingual populations and those with diverse educational attainment, its use in detecting and/or estimating cognitive decline, especially among adults with SCD (most of whom are younger), is still unclear. Recruitment for a future study is ongoing to ascertain the reliability and to further validate the ability of the RUDAS to detect clinically significant cognitive dysfunction in adult patients with SCD.

### Limitations

This study has several limitations. First, its cross-sectional design precluded our ability to use the RUDAS to detect the development of dementia at this time. Second, major vascular cognitive disorder diagnosis according to the International Society for Vascular Behavioral and Cognitive Disorders guidelines requires a decline from previous function; therefore, we can only suspect such a diagnosis.^37^ A comprehensive clinical assessment, including the implication for independence and the subjective experience, is needed to confirm the diagnosis. Given that access to comprehensive clinical assessment for dementia is limited because of its specialized and cumbersome nature, the RUDAS can potentially fill the niche as a screening tool that allows the identification and channeling of patients with suspected dementia for formal assessment. Certain deficits observed in the RUDAS testing may reflect the neurodevelopmental impact of SCD early on in life, but the increased prevalence between age groups suggests the presence of an additional neurodegenerative process during adulthood. Third, the optimal threshold to flag a patient with SCD as having likely dementia or mild NCD will need to be established in future studies. Fourth, selection bias was also a potential concern. Convenience sampling was applied to recruit successive patients from ambulatory care settings in CHUM and UHN. Patients with dementia may have difficulties attending clinic visits. However, for these patients, clinicians may already have sufficient suspicion and indirect evidence of cognitive dysfunction for referral to the appropriate resources, and a screening tool may not be needed to unmask cognitive dysfunction. Fifth, although GFR was associated with the RUDAS scores, we cannot exclude the presence of additional confounders, such as diabetes, heart failure, medications, and other unknown factors.

## Conclusions

Cognitive dysfunction screening using the RUDAS revealed a high prevalence of suspected dementia in adult patients with SCD that was associated with worsening kidney function and age. The results of this study suggest that all adult patients with SCD, regardless of age, disease severity, and SCD genotype should be screened for cognitive dysfunction. A study is underway to ascertain the reliability and validity of the RUDAS to detect clinically significant cognitive dysfunction in adult patients with SCD.

## Notes

Supplement. **eTable 1.** Correction for Multiplicity of Analyses Presented in Table 3 **eTable 2.** Prediction of RUDAS Z-Scores Using Multiple Regression (After Multiple Imputation [n = 5]) **eTable 3.** Results from 5 Iterations of Multiple Imputation of All Missing Variables in the Study
