# Supplementary material for: Screening for Cognitive Dysfunction Using the Rowland Universal Dementia Assessment Scale in Adults With Sickle Cell Disease
Source: JAMA Netw Open. 2021 May 13;4(5):e217039. doi: 10.1001/jamanetworkopen.2021.7039 (PMC8120324; doi:10.1001/jamanetworkopen.2021.7039)
Supplement: Supplement. — eTable 1. Correction for Multiplicity of Analyses Presented in Table 3 eTable 2. Prediction of RUDAS Z-Scores Using Multiple Regression (After Multiple Imputation [n = 5]) eTable 3. Results from 5 Iterations of Multiple Imputation of All Missing Variables in the Study [file jamanetwopen-e217039-s001.pdf]

Supplementary Online Content

Forté S, Blais F, Castonguay M, et al. Screening for cognitive dysfunction using the Rowland Universal Dementia Assessment Scale in adults with sickle cell disease. *JAMA Netw Open*. 2021;4(5):e217039. doi:10.1001/jamanetworkopen.2021.7039

eTable 1. Correction for Multiplicity of Analyses Presented in Table 3

eTable 2. Prediction of RUDAS Z-Scores Using Multiple Regression (After Multiple Imputation [n=5])

eTable 3. Results from 5 Iterations of Multiple Imputation of All Missing Variables in the Study

This supplementary material has been provided by the authors to give readers additional information about their work.

© 2021 Forté S et al. *JAMA Network Open.*

**eTable 1.** Correction for Multiplicity of Analyses Presented in Table 3<br><br>The Holm's correction was applied.		
**Characteristics**	**Uncorrected p-value**	**Corrected p-value**
Biologic determinants		
**Sex**	0.30	1.00
**Age**	<0.001	0.003
**SCD genotype**	0.95	1.00
**Alpha thalassemia mutation status**	0.34	1.00
**Language and social determinants of health**		
**Site**	0.43	1.00
**Language**	0.45	1.00
**Mean household income**	0.67	1.00
**Highest level of education**	0.02	0.38
**Occupation**	<0.001	<0.001
**SCD-related complications**		
**History of acute chest syndrome**	0.23	1.00
**Self-reported SCD related pain**	0.93	1.00
**Sepsis**	0.98	1.00
**Stroke**	0.71	1.00
**Sebastiani SCD severity score**	0.62	1.00
**Comorbid conditions**		
**Depression**	0.09	1.00
**Anxiety disorder**	0.38	1.00
**Systolic blood pressure**	0.03	0.72
**Laboratory parameters**		
**Bilirubin**	0.20	1.00
**Lactate dehydrogenase**	0.35	1.00
**Hematocrit**	0.49	1.00
**Reticulocyte count**	0.01	0.31
**Mean corpuscular volume**	0.99	1.00
**Fetal hemoglobin**	0.86	1.00
**White blood cell count**	0.38	1.00
**Glomerular filtration rate**	<0.001	<0.001
**Disease-modifying therapies**		
**Prior blood transfusion**	0.49	1.00
**Hydroxyurea**	0.57	1.00
**Regular exchange transfusion**	0.22	1.00
Abbreviation: SCD=sickle cell disease.		

© 2021 Forté S et al. *JAMA Network Open.*

eTable 2. Prediction of RUDAS Z-Scores Using Multiple Regression (After Multiple Imputation [n=5])

Unstandardized estimates, confidence intervals (CI), standard errors (SE), standardized estimates, t-values and p-values are listed for each of the 12 variables of the multiple regression model.

	**Unstandardized estimate and CI**	**SE**	**Standardized estimate**	***t***	**p-value**
**(Intercept)**	-1.07 [-2.31;0.17]	0.63	0.00	-1.69	0.09
**Age**	-0.01 [-0.02;0.00]	0.01	-0.17	-2.15	0.03
**Sex**	0.21 [-0.06;0.47]	0.13	0.11	1.56	0.12
**Highest level of education**	0.09 [0.02;0.16]	0.04	0.15	2.44	0.02
**Income**	0.00 [0.00;0.00]	0.00	0.06	1.00	0.32
**Genotype**	-0.01 [-0.13;0.12]	0.06	-0.01	-0.08	0.94
**Stroke**	-0.05 [-0.45;0.36]	0.21	-0.02	-0.24	0.81
**Depression**	0.28 [-0.16;0.73]	0.23	0.08	1.24	0.22
**Anxiety**	-0.06 [-0.59;0.46]	0.27	-0.02	-0.24	0.81
**Pain**	-0.16 [-0.45;0.12]	0.15	-0.07	-1.12	0.26
**Hematocrit**	-1.53 [-4.06;1.01]	1.28	-0.09	-1.19	0.23
**GFR**	0.01 [0.01;0.02]	0.00	0.32	4.22	<0.01
**Reticulocytes**	0.28 [-0.01;0.57]	0.15	0.13	1.88	0.06

Abbreviations: CI=confidence interval, GFR=glomerular filtration rate, RUDAS= Rowland Universal Dementia Assessment Scale, SE= standard errors.

eTable 3. Results from 5 Iterations of Multiple Imputation of All Missing Variables in the Study

Imputed data set	$R^2(F^2)$	Adjusted $R^2(F^2)$	F	P value	Degrees of freedom	Number of observations
**1**	0.24(0.32)	0.20(0.25)	5.49	<0.001	12	218
**2**	0.24(0.32)	0.20(0.25)	5.49	<0.001	12	218
**3**	0.24(0.32)	0.20(0.25)	5.49	<0.001	12	218
**4**	0.24(0.32)	0.20(0.25)	5.49	<0.001	12	218
**5**	0.24(0.32)	0.20(0.25)	5.49	<0.001	12	218

© 2021 Forté S et al. *JAMA Network Open.*
